# Supplementary material for: Transcriptomic and Functional Comparison of Cells Isolated From Healthy and Degenerated Ovine Intervertebral Discs
Source: J Cell Mol Med. 2026 Jan 22;30(2):e71026. doi: 10.1111/jcmm.71026 (PMC12825213; doi:10.1111/jcmm.71026)
Supplement: Supplementary file 1 — Figure S1: Relative to Main Figure 1. (A) Representative Alcian Blue staining images. Scale bar: 10 mm. (B) Cell morphology at passage P1. Scale bar: 100 μm. (C) Cell count after cell isolation. (D) Cellular doubling time between passage P0 and P1. n.s., not significant; *p < 0.05. p‐values were calculated using Mann Whitney test; N = 4 young and N = 4 old animals. Figure S2: Schematic bioinformatic analysis pipeline. (A) From primary to secondary analysis pipeline. Figure S3: Relative to main Figure 2. (A) Heatmap showing sample clustering based on matrix‐related transcripts expression from DIPPER analysis [35]. (B) Volcano plot of differential gene expression in NPC versus AFC from young and (C) old animals using the Wald test. (D) Geneset Enrichment Analysis (GSEA) AFC versus NPC samples using the permutation test. (E) UMAP of scRNA‐seq human dataset (GSE199866) split between one healthy and one IVDD intervertebral disc, cells from NP tissue are highlighted in turquoise and from AF tissue in orange. Figure S4: Relative to main Figure 3. (A) UMAP of scRNA‐seq human dataset (GSE199866) used as basis for bulk RNA‐seq deconvolution combining AFC and NPC transcriptome, split between one healthy and one degenerated intervertebral disc. Cellular states were annotated using literature consensus. (B) Proportion of the different cellular states present in healthy and IVDD condition. (C) Dendrogram plot showing hierarchical clustering of the 12159 genes in 14 modules after merging, a different colour represents each module. (D) Dendrogram and heatmap generated with the module eigengenes for the 14 modules identified. Significant association between modules and origin of the cells are highlighted below (Yng: young). p‐values were calculated with the Student asymptotic p‐value for correlation. Figure S5: Relative to main Figure 4. NPC from young and aged ovine disc share common senescence and mitochondrial energetic responses to stress‐related stimuli. (A) IL6 and MMP3 transc [file JCMM-30-e71026-s004.pdf]

Transcriptomic and functional comparison of cells isolated from healthy and degenerated ovine intervertebral discs

Paul Humbert[1], Lucie Danet[1], Emmaëlle Carrot[1], Floriane Etienne[1,2], Boris Halgand[1], Frédéric Blanchard[1], Claire Vinatier[1], Jérôme Guicheux[1], Marion Fusellier[1,2], Catherine Le Visage[1], Romain Guiho[1,*]

[1] Nantes Université, Oniris, CHU Nantes, INSERM, Regenerative Medicine and Skeleton, RMeS, UMR 1229, F-44000 Nantes, France

[2] Department of Diagnostic Imaging, CRIP, ONIRIS, College of Veterinary Medicine, Food Science and Engineering, Nantes, France

*** Corresponding author:**

Romain Guiho, Inserm UMRS 1229-RMeS Regenerative Medicine & Skeleton, School of dental medicine, 1 place Alexis Ricordeau, 44042 Nantes cedex 1, France.
romain.guiho@univ-nantes.fr

Supplementary Methods

Supplementary Figures

Supplementary Tables legends

Supplementary Methods

Extended Sample Collection Protocol

Lumbar IVDs were collected from four 6-month-old female lambs and four female sheep between 7-8 years-old (Vendée breed, GAEC HEAS, Les Rabelais, Ligné, France) in the accredited Centre of Research and Pre-Clinical Investigations (ONIRIS, National Veterinary School of Nantes). The sheep received a bolus hypocoagulant dose of heparin (50 IU/kg) and were euthanized with an overdose of barbiturates (60 mg/kg) following the best practices and legislation (European Directive 2010/63/EU). Post-euthanasia, MRI of the lumbar spine was performed to obtain sagittal T2-weighted images (TE: 86 ms, TR: 3,000 ms; slice thickness: 3 mm), using a 1.5 T MRI scanner (Magnetom Essenza, Siemens Medical Solutions). OsiriX 9 software (Osirix Foundation) was used to analyse MRI images, and each lumbar IVD was scored using the Pfirrmann grading system. Under aseptic conditions, the lumbar spines were extracted via a posterior approach, and the IVDs were individualized with part of the vertebral bodies using an oscillating saw. The sections were then transported to the cell culture room in sterile media.

Extended Cell Isolation and Culture Protocol

Five lumbar IVDs per donor were dissected from the vertebral bodies, AF and NP were separated and diced into pieces. Tissue fragments were rinsed three times in HBSS (L0606, Biowest) with 2% penicillin/streptomycin (P/S; 15070-063, Gibco) for 2 minutes. The extracellular matrix was then digested with 0.05% hyaluronidase (H4272, Sigma-Aldrich) in HBSS at 37 °C for 15 minutes. After two rinses with HBSS, 15–20 mL of 0.2% trypsin (T9935, Sigma-Aldrich) in HBSS were added to each sample and incubated at 37 °C for 30 minutes. After two additional rinses with HBSS, 15–20 mL of 0.25% collagenase (C5138, Sigma Aldrich) were added in complete culture medium, i.e. DMEM high glucose and pyruvate (31966-021, Gibco) supplemented with 10% foetal bovine serum (FBS eurobio scientific CVFSCF00-01, Lot n° S76214), 1% P/S, and 0.1% Amphotericin B (15290, Gibco), and the mixtures were incubated at 37 °C overnight. The recovered suspension was filtered through a 70-µm-pore filter and centrifuged for 5 minutes at 300 g. The cells were counted and seeded at 10 000 cells /cm² in T75 flasks (Sarstedt). AF and NP cells were grown at 37 °C and 5% CO_2.

Quantitative RT-PCR Analysis of IL-6 and MMP3 Expression in NP Cells

NP cells at P3 were seeded in 6-well plates at 100,000 cells per well. After 3 days, the culture medium was changed after a PBS wash to (1) complete culture medium, (2) complete culture medium supplemented with ovine IL-1β (10ng/mL), or (3) serum-free culture medium. After 24h, total RNA was extracted using the Nucleospin RNA XS kit (Macherey-Nagel, 740902) according to the manufacturer's instructions. RNA yield was measured using NanoDrop 1000 Spectrophotometer (Thermo Scientific). Reverse transcription was performed using the Verso cDNA Synthesis Kit (Thermo Scientific, AB1453B) with a mix of Anchored Oligo(dT) and Random Hexamers. Real-time polymerase chain reaction (PCR) was performed using specific *ovis aries* primers (5' -> 3': IL6_Forward: CCTGTCCACTGGGCACATAA; IL6_Reverse: GTTCAAGCCGCATAGCCATT; MMP3_Fw: GTGTGATCCTGCCTTGTCCT; MMP3_Rev: CCGCCAAAAATGTCTGCCTTT; housekeeping gene: GAPDH_Fw: TCCTGCCCCACCTCCACCAC; GAPDH_Rev: GGGCTCCCTAAGCCCCTCCC) with SYBR Select Master Mix (Applied Biosystems, 4472908) on the CFX96 Touch Real-time PCR Detection System (Bio-Rad).

Supplementary Figures

Supplementary Figure S1

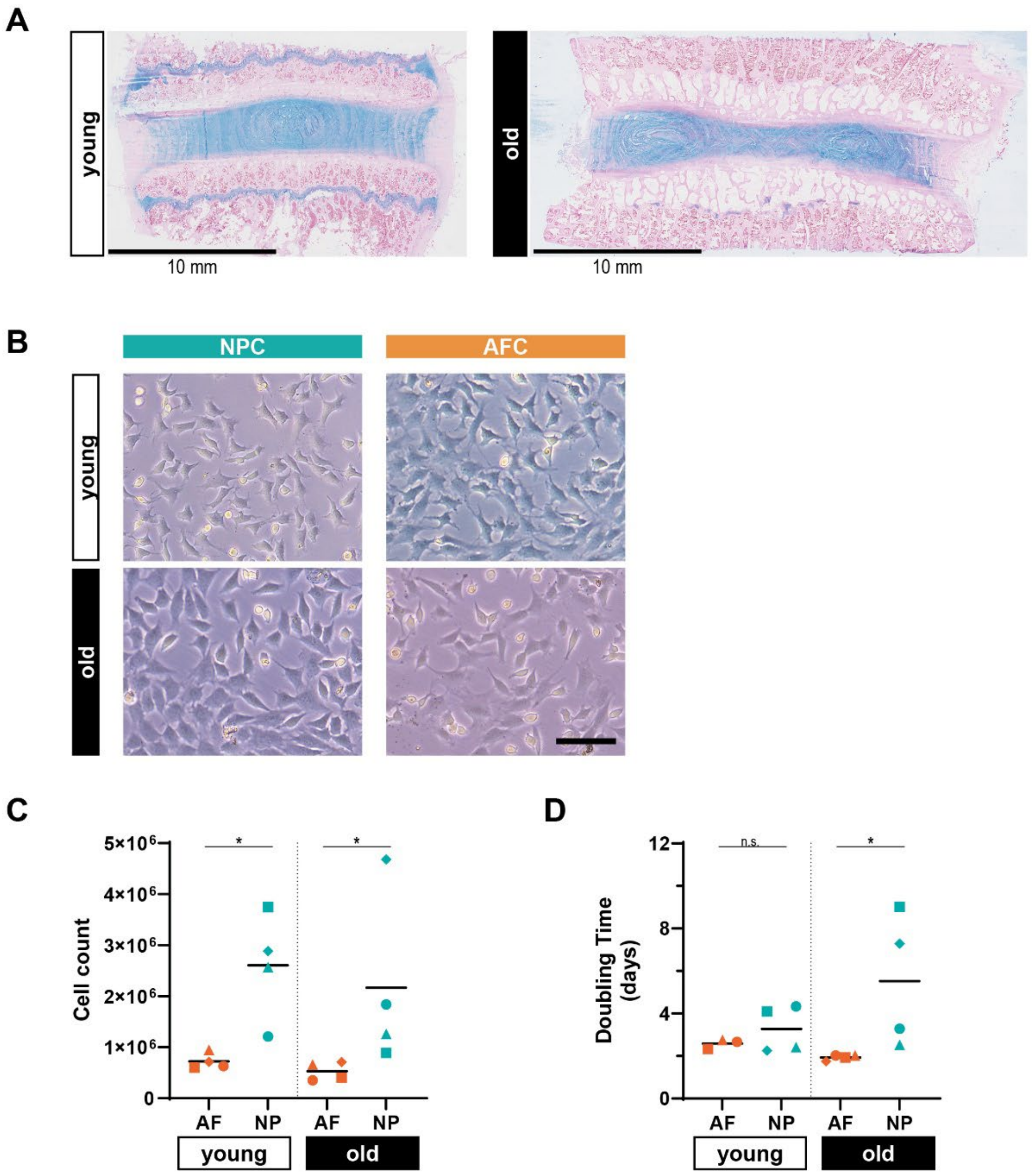

Supplementary Figure S1 – Relative to Main Figure 1. A. Representative Alcian Blue staining images. Scale bar: 10 mm. **B.** Cell morphology at passage P1. Scale bar: 100µm. **C.** Cell count after cell isolation. **D.** Cellular doubling time between passage P0 and P1. n.s.: not significant; *: $p<0.05$. p-values were calculated using Mann Whitney test; N=4 young and N=4 old animals.

Supplementary Figure S2

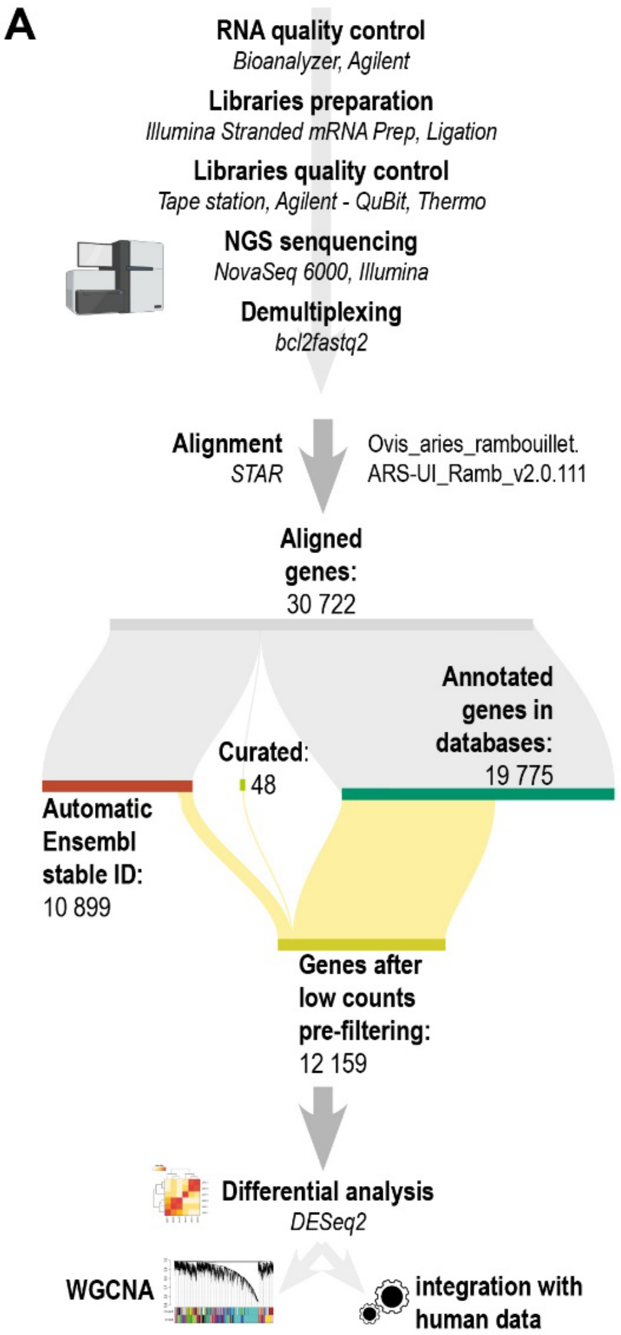

Supplementary Figure S2. Schematic bioinformatic analysis pipeline. A. From primary to secondary analysis pipeline.

Supplementary Figure S3

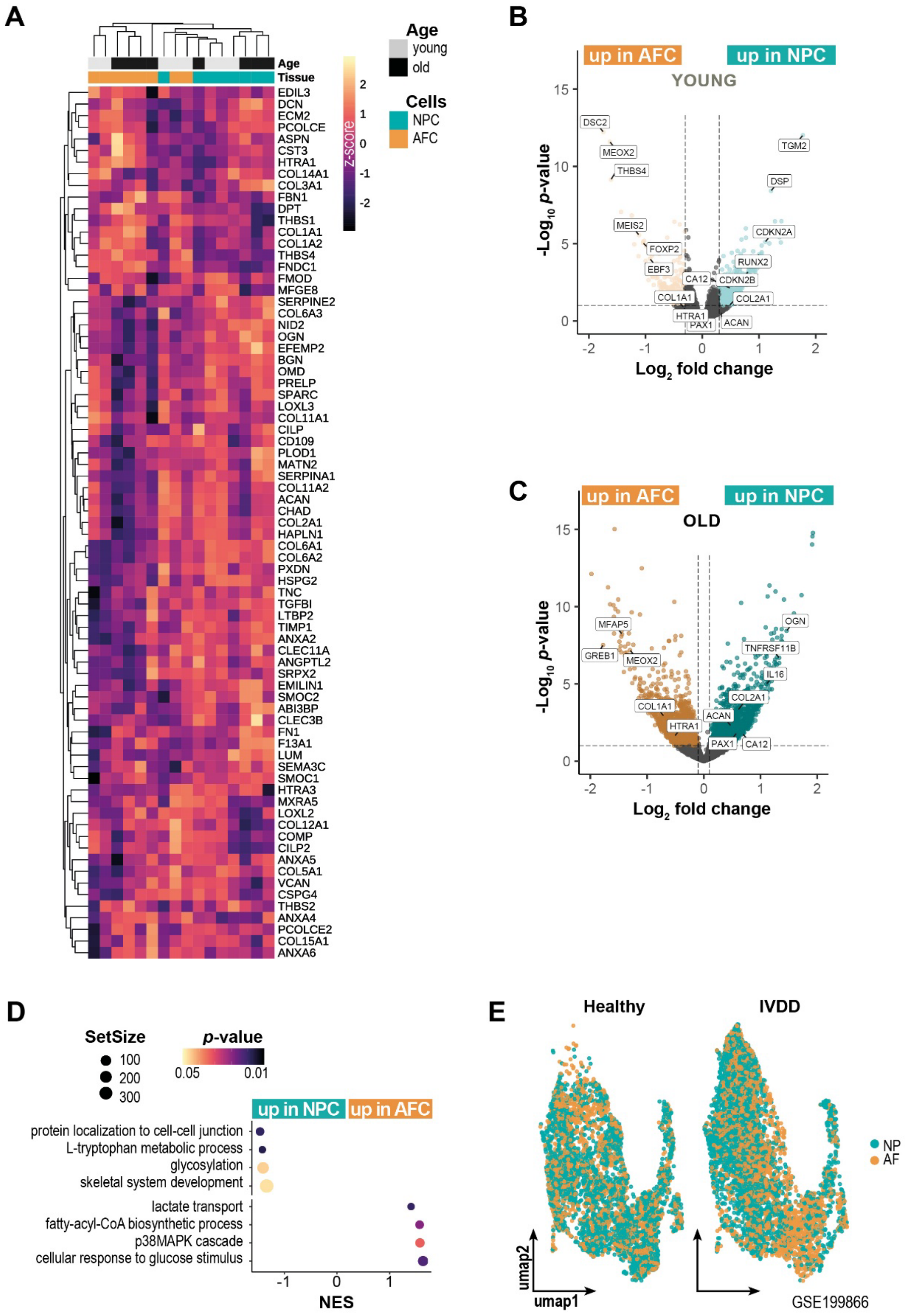

Supplementary Figure S3 – *Relative to Main Figure 2*. A. Heatmap showing sample clustering based on matrix-related transcripts expression from DIPPER analysis (Tam et al.

2020). **A**. Volcano plot of differential gene expression in NPC versus AFC from young and **B.** old animals using the Wald test. **D.** Geneset Enrichment Analysis (GSEA) AFC versus NPC samples using the permutation test. **E.** UMAP of scRNA-seq human dataset (GSE199866) split between one healthy and one IVDD intervertebral disc, cells from NP tissue are highlighted in turquoise and from AF tissue in orange.

Supplementary Figure S4

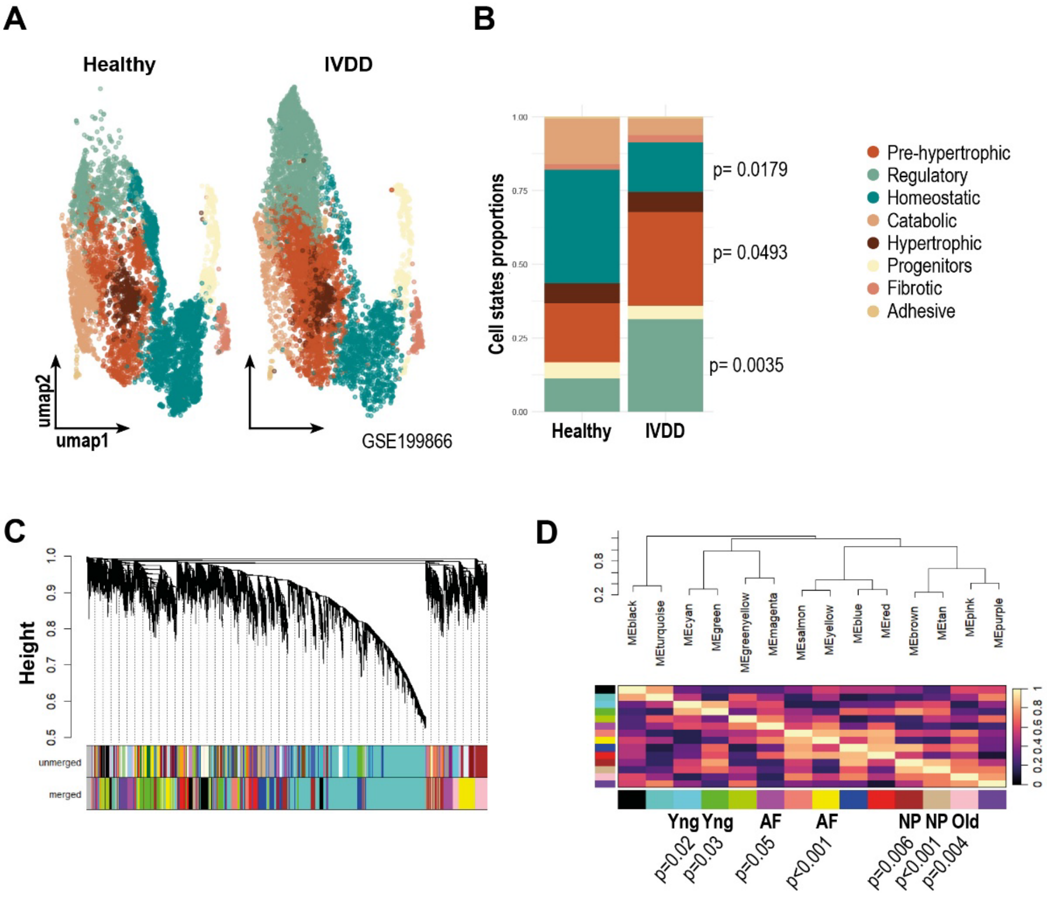

Supplementary Figure S4 – *Relative to Main Figure 3*. A. UMAP of scRNA-seq human dataset (GSE199866) used as basis for bulk RNA-seq deconvolution combining AFC and NPC transcriptome, split between one healthy and one degenerated intervertebral disc. Cellular states were annotated using literature consensus. **B.** Proportion of the different cellular states present in healthy and IVDD condition. **C.** Dendrogram plot showing hierarchical clustering of the 12159 genes in 14 modules after merging, a different colour represents each module. **D.** Dendrogram and heatmap generated with the module eigengenes for the 14 modules identified. Significant association between modules and origin of the cells are highlighted below (Yng: young). p-values were calculated with the Student asymptotic p-value for correlation.

Supplementary Figure S5

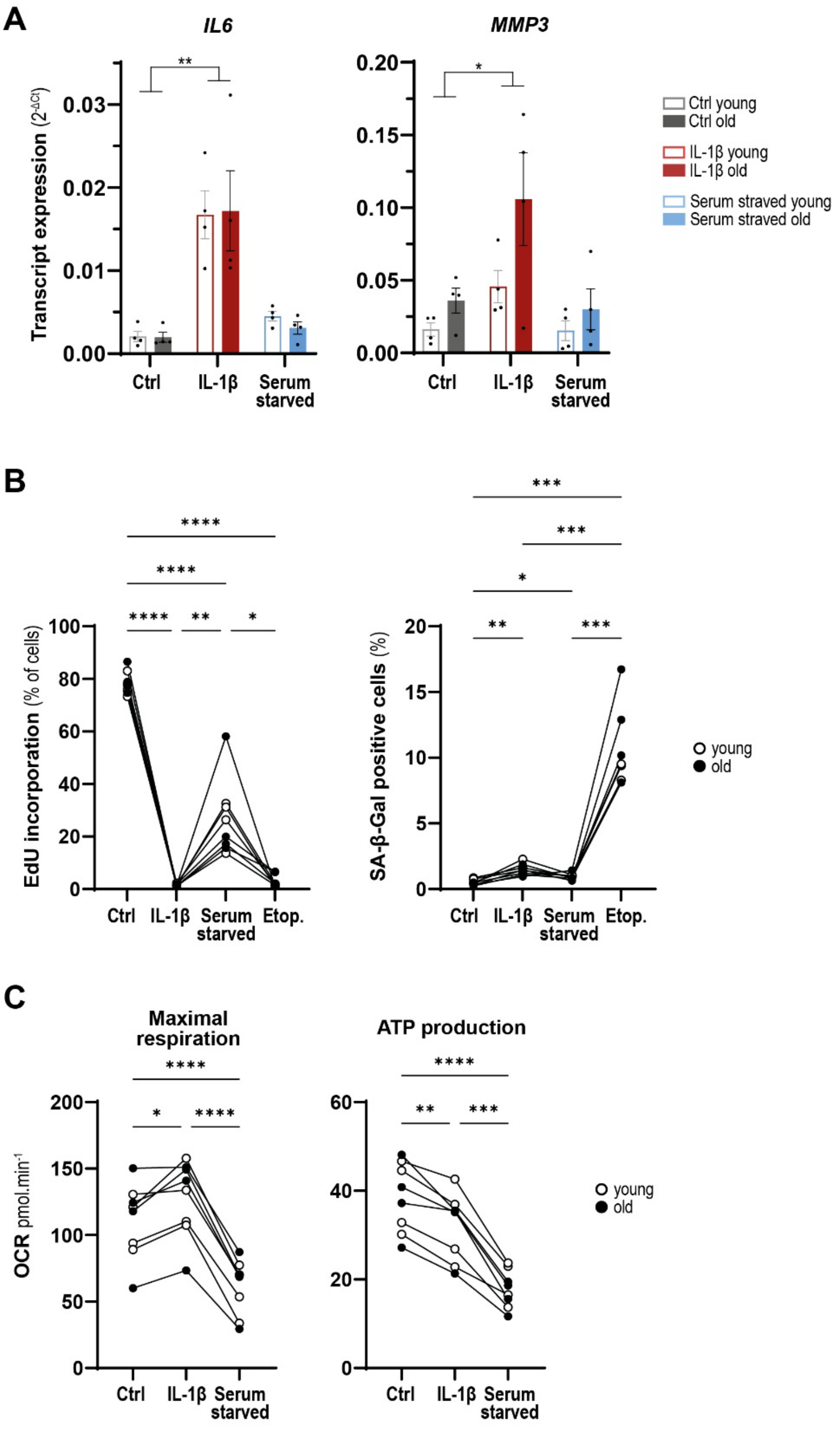

Supplementary Figure S5 – ***Relative to Main Figure 4*****. NPC from young and aged ovine disc share common senescence and mitochondrial energetic responses to stress-related stimuli. A.** IL6 and MMP3 transcript expression by RT-qPCR of NPC from young and old animals following 24h of IL-1 β treatment or serum starvation. **B.** Quantification of % of NPC from young and old animals positive for EdU incorporation and SA-β-Gal staining. **C.**

Quantification of maximal respiration and ATP production. *:$p<0.05$; **:$p<0.01$; ***:$p<0.001$; ****:$p<0.0001$. p-values were calculated using the Repeated Measures ANOVA test, and Tukey's post-hoc test; N=8 animals.

Supplementary Tables legends

Supplementary Table 1. Differential Expression Results between NPC from young and old animals. Standard differential expression analysis results using DESeq2 pipeline. baseMean= mean of normalized read count across all samples. log2 fold change= log2 Fold change between groups, positive indicates upregulated in NPC isolated from old animals, padj= p-value adjusted for multiple testing using Benjamini-Hojberg correction.

Supplementary Table 2. Differential Expression Results between AFC from young and old animals. Standard differential expression analysis results using DESeq2 pipeline. baseMean= mean of normalized read count across all samples. log2 fold change= log2 Fold change between groups, positive indicates upregulated in AFC isolated from old animals, padj= p-value adjusted for multiple testing using Benjamini-Hojberg correction.

Supplementary Table 3. Differential Expression Results between NPC and AFC isolated from young animals. Standard differential expression analysis results using DESeq2 pipeline. baseMean= mean of normalized read count across all samples. log2 fold change= log2 Fold change between groups, positive indicates upregulated in NPC, padj= p-value adjusted for multiple testing using Benjamini-Hojberg correction.

Supplementary Table 4. Differential Expression Results between NPC and AFC isolated from old animals. Standard differential expression analysis results using DESeq2 pipeline. baseMean= mean of normalized read count across all samples. log2 fold change= log2 Fold change between groups, positive indicates upregulated in NPC, padj= p-value adjusted for multiple testing using Benjamini-Hojberg correction.

Supplementary Table 5. List of the top-30 genes associated with WGCNA modules. List of the 30 genes most associated with the different modules identified by WGCNA, ranked by importance in the module. The modules included are the modules associated with AFC (Yellow and Magenta modules), with NPC (Tan and Brown modules), with young and (Cyan and Green modules) and with old age (Pink module).
